# Myasthenic symptoms in anti-low-density lipoprotein receptor-related protein 4 antibody-seropositive amyotrophic lateral sclerosis: two case reports

**DOI:** 10.1186/s12883-016-0758-1

**Published:** 2016-11-18

**Authors:** Hisashi Takahashi, Yu-ichi Noto, Naoki Makita, Yukie Kushimura-Okada, Ryotaro Ishii, Akihiro Tanaka, Tomoyuki Ohara, Shunya Nakane, Osamu Higuchi, Masanori Nakagawa, Toshiki Mizuno

**Affiliations:** 1 Department of Neurology, Kyoto Prefectural University of Medicine Graduate School of Medical Science, 465 Kajii-cho, Kamigyo-ku, Kyoto, 602-0841 Japan; 2 Department of Neurology, National Hospital Organization Maizuru Medical Center, Maizuru, Japan; 3 Department of Neurology, Graduate School of Medical Sciences, Kumamoto University, Kumamoto, Japan; 4 Department of Clinical Research, Nagasaki Kawatana Medical Center, Kawatana, Japan; 5 Director of North Medical Center, Kyoto Prefectural University of Medicine, Kyoto, Japan

**Keywords:** Case report, Amyotrophic lateral sclerosis, Myasthenic symptom, Myasthenia gravis, Anti-LRP4 antibody, Immunotherapy, Luciferase immunoprecipitation systems

## Abstract

**Background:**

Myasthenic symptoms can be present in patients with amyotrophic lateral sclerosis (ALS). These symptoms have been considered to be caused by the degeneration of distal motor neurons and the neuromuscular junction (NMJ). Recent studies suggested that antibody to low-density lipoprotein receptor-related protein 4 (LRP4) was a pathogenic agent of myasthenia gravis (MG), and it was also detected in ALS patients.

**Case presentation:**

Patient 1: A 58-year-old Japanese man developed progressive weakness and subsequent myasthenic symptoms including oculomotor disturbance. Clinical examination and electrophysiological studies confirmed upper and lower motor neuron involvement and NMJ dysfunction, and anti-LRP4 antibody was detected in his serum. A series of immunotherapies, including steroid pulse therapy, intravenous immunoglobulin, and plasmapheresis, was performed, and the myasthenic symptoms partially improved. The titer of anti-LRP4 antibody subsequently decreased. However, the therapeutic effect was transient, and ALS symptoms progressed. His clinical findings fulfilled the criteria of probable ALS using the Awaji criteria. Patient 2: A 74-year-old Japanese man suffered from progressive weakness of all limbs and dropped head in the evening. He complained of diplopia with a lateral horizontal gaze. Probable ALS was diagnosed because of the upper and lower motor neuron signs, whereas anti-LRP4 antibody was detected. Several immunotherapies were administered, and the myasthenic symptoms partially responded to each therapy. However, the truncal muscle weakness progressed, and he died of respiratory failure.

**Conclusion:**

We report two anti-LRP4 antibody-seropositive ALS patients with myasthenia who were not typical of ALS patients, and showed partial responses to immunotherapies. The anti-LRP4 antibody-seropositive status may influence developing ALS and cause additional ALS symptoms.

## Background

Amyotrophic lateral sclerosis (ALS) is a fatal neurodegenerative disease in which the selective degeneration of the upper and lower motor neuronal system causes muscle weakness, atrophy, cramp, and fasciculation combined with spasticity. The mechanism of neurodegeneration in sporadic ALS remains unclear. Although various hypotheses have been put forward, including glutamate-mediated excitotoxicity, protein aggregation, apoptosis, astrocyte dysfunction, mitochondrial dysfunction, increased oxidative stress, and axonal ion channel dysfunction, an autoimmune mechanism has been proposed [1].

Patients with ALS occasionally present with myasthenia-like symptoms such as increased muscle fatigability. Myasthenia-like symptoms are thought to be attributed to dysfunction of the neuromuscular junction (NMJ) due to distal collateral branching after axonal loss [2, 3]. On the other hand, symptoms in patients with myasthenia gravis (MG) are caused by autoantibodies to the NMJ. There are two established pathogenic autoantibodies for MG: an anti-acetylcholine receptor (AchR) antibody, and a muscle-specific tyrosine kinase (MuSK) antibody. Both AchR and MuSK are essential components of the NMJ, and their dysfunction and injury due to autoantibodies cause NMJ dysfunction, leading to myasthenia [4].

Recently, an autoantibody to low-density lipoprotein receptor-related protein 4 (LRP4) was detected in the serum of some MG patients [5, 6]. LRP4 is a component of the NMJ as well as AChR and MuSK and is also indispensable for NMJ formation and maintenance [7, 8]. Moreover, it has been demonstrated that anti-LRP4 antibody is a directly pathogenic agent causing MG [9]. Regarding ALS, Tzartos et al. reported that anti-LRP4 antibodies were detected in the serum and cerebrospinal fluid (CSF) of patients with ALS, and suggested that the antibody may be more broadly associated with damage to LRP4-expressing tissues, such as motor neurons and the NMJ [10]. However, the pathogenic role of anti-LRP4 antibodies remains unclear in ALS. Here, we describe two anti-LRP4 antibody-seropositive ALS patients with myasthenia.

## Case Presentation

**Fig. 1:**
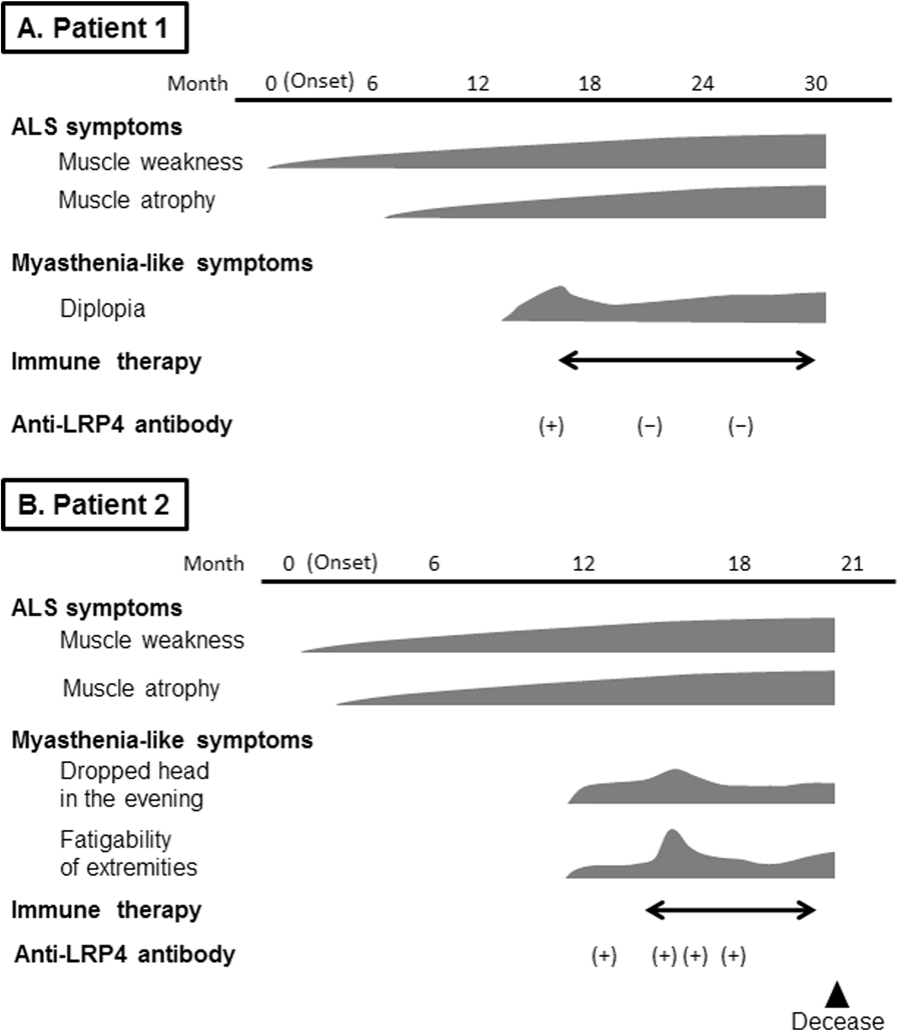
The clinical course of anti-low-density lipoprotein receptor-related protein 4 antibody-seropositive amyotrophic lateral sclerosis patients with myasthenic symptoms. **a** patient 1, **b** patient 2
- Patient 2
  A 74-year-old Japanese right-handed man was admitted to hospital with weakness of the right hand and both legs and a dropped head. Ten months before admission, he was aware of clumsiness and suffered painful cramps of his right hand. His dropped head aggravated in the evening. He had no previous history of diplopia or dysphagia. His previous medical and family histories were unremarkable.
  On neurological examination, he presented with diplopia with a horizontal gaze to the right side for over 15 s. He had no dysarthria, and no tongue abnormality. His grip strength on the right side was lower than that on the left (28 and 32 kg, respectively). He had mild weakness of MRC grade 4/5 and atrophy of the right upper limb, neck, paraspinal, and abdominal muscles. Fasciculations were extensively observed in the limbs and truncal muscles. The deep tendon reflexes were increased in the upper and lower limbs. Sensory examination was normal. The edrophonium test demonstrated the improvement of diplopia, but muscle weakness remained unchanged. NCS and RNST revealed decreased compound muscle action potential amplitudes in the right abductor pollicis brevis (APB) and FDI muscles with normal conduction velocities and a 10.6% decrement in the right ADM muscle on 3-Hz stimulation, respectively. Increased jitter was also seen in s-SFEMG recorded at the right EDC muscle. In EMG performed on the right side, FPs were detected in the trapezius, paraspinal, and upper and lower limbs muscles, and fibrillation potentials or positive sharp waves were identified in the FDI, paraspinal, and tibialis anterior muscles. Reduced recruitment with high-amplitude motor unit potentials was also observed in the trapezius, FDI, and vastus lateralis muscles. On MEP testing of the AH muscles, the central motor conduction time was prolonged on the right side. In the serological test, anti-AChR and anti-MuSK antibodies were negative. Brain MRI was normal, and whole-spine MRI showed mild lumbar spondylosis.
  The patient was diagnosed with probable ALS using the Awaji criteria, whereas the fluctuation of his dropped head symptom and diplopia after loading were not consistent with the diagnosis of ALS, but suggested a diagnosis of MG. A few weeks after admission, anti-LRP4 antibody in serum was positive (A.I.: 1.50) based on LIPS.
  As in patient 1, steroid pulse therapy was conducted. A few days after the therapy, the frequency of clinical fasciculations and FPs on EMG decreased. However, fatigability of the upper limb and neck muscles worsened, and decrement at the right trapezius muscle was newly detected in the follow-up RNST. The QMG score worsened from 9 to 12 points. This clinical course was thought to represent an initial escalation of MG at that time. Subsequently, we conducted plasmapheresis therapy. Fatigability improved, and the decrease in the RNST was reduced after this therapy, but auto-LRP4 antibody remained positive (A.I.: 1.15). Six months after admission, dyspnea due to respiratory muscle weakness developed, and he died 7 months after admission. Clinical examination just prior to his death revealed mild muscle weakness of MRC grade 4/5 of the neck and limbs, without tongue muscle atrophy or dysphagia. His clinical course is shown in Fig. 1b.

- Patient 1
  The patient was a 58-year-old, right-handed man who was admitted to our hospital. At 57 years of age, he developed dysarthria and weakness of the fingers on the right hand. A few months prior to admission, he started to experience leg muscle cramps and occasionally noticed diplopia during lateral gaze. The severity of diplopia and dysarthria fluctuated within a day and on a daily basis. He had a history of cervical spondylosis with no surgical treatment. His family history was unremarkable.
  On neurological examination, the abducent ocular movement was incomplete bilaterally. Moreover, he had dysarthria and mild tongue atrophy with fasciculation. His hand muscles showed atrophy with weakness on the right side, with Medical Research Council (MRC) grade 4/5. Although there was no apparent atrophy of other muscles, fasciculations were observed bilaterally in the upper and lower limbs and trunk muscles. The grip strength on the right side was weaker than that on the left (34 and 35 kg, respectively). He could not maintain a raised head for 90 s in a supine position because of the progressive fatigability of neck muscles. Sensory examination revealed nothing of note. Deep tendon reflexes were normal, whereas the Wartenberg reflex was present bilaterally. The edrophonium test revealed moderate improvements of his dysarthria and ocular movement impairment, whereas the hand muscle weakness on the right side showed no improvement. A routine nerve conduction study (NCS) revealed nothing of note except for prolonged distal latency in the right median nerve, and repetitive nerve stimulation testing (RNST) of the ulnar, facial, and accessory nerves also yielded results within normal limits. On electromyography (EMG), fibrillation potentials and polyphasic motor unit potentials were detected in the right first dorsal interossei (FDI) and right biceps brachii muscles, and fasciculation potentials (FPs) were observed in the right trapezius, FDI, biceps brachii, vastus lateral, and tibialis anterior (TA) muscles. Stimulated-single fiber electromyography (s-SFEMG) showed increased jitter in the right extensor digitrum communis (EDC) muscle. On motor-evoked potential (MEP) testing recorded at the abductor hallucis (AH) muscle, the motor action potential was typically evoked on the left but not on the right side. Serological examination revealed that anti-AChR and anti-MuSK antibodies were negative. Head magnetic resonance imaging (MRI) showed a laminar high-intensity lesion along the left motor cortex on susceptibility-weighted imaging, which indicated iron deposition. Cervical MRI revealed cervical spondylosis at the C4/5/6/7/Th1 level, as well as mild compression of the spinal cord at the C4/5 level with no myelomalacia.
  Abundant fasciculations in EMG and MEP and MRI findings suggesting upper motor neuron dysfunction indicated a diagnosis of ALS, whereas his ocular movement dysfunction, fatigability of neck muscles, and results of the edrophonium test were consistent with a diagnosis of MG. A few weeks following admission, anti-LRP4 antibody was positive (antibody index (A.I.): 1.08) using the luciferase immunoprecipitation systems (LIPS) [5]. In this report, the antibody levels are expressed as A.I., calculated as follows:
  $$ \mathrm{A}.\mathrm{I}. = \left[\mathrm{measurement}\ \mathrm{value}\ \mathrm{of}\ \mathrm{the}\ \mathrm{sample}\ \mathrm{serum}\ \left(\mathrm{relative}\ \mathrm{luminescence}\ \mathrm{units}\right)\right]/\left[\mathrm{cut}-\mathrm{off}\ \mathrm{value}\ \left(\mathrm{relative}\ \mathrm{luminescence}\ \mathrm{units}\right)\right]. $$
  The normal value established for healthy individuals is <1.0 [5]. Therefore, we started immunotherapy while considering his condition to be immune-mediated. Initially, steroid pulse therapy (methylprednisolone at 1000 mg per day for 3 days) was conducted. The dysarthria and neck muscle fatigability partially improved, and the frequency of fasciculations decreased throughout his body. The quantitative myasthenia gravis (QMG) score improved from 11 points before to 6 points after treatment [11]. Four weeks after the treatment, his symptoms progressed again. Subsequently, treatments with oral corticosteroids, intravenous immunoglobulin, and plasmapheresis therapy were performed. The ocular movements, diplopia, and dysarthria improved mildly and transiently by each treatment, and the titer of anti-LRP4 antibody normalized after these treatments (A.I.: 0.67). However, all immunotherapies had limited effects, and weakness and fatigability progressed gradually. Six months after admission, muscle atrophy and weakness of the left hand and both legs developed, and his grip strength decreased (right: 21 kg, left: 11 kg). In addition, a bilateral Babinski sign appeared 12 months after admission. A follow-up EMG study showed the spread of active denervations of the limbs. At that time, he fulfilled the diagnostic category of probable ALS using the Awaji criteria [12, 13]. The clinical course is shown in Fig. 1a.

## Conclusion

We encountered two anti-LRP4 antibody-seropositive patients who were consistent with the diagnostic criteria and clinical course of ALS with some myasthenic symptoms that were partially improved by immunotherapies. Although Mulder et al. reported four ALS patients with a myasthenia-like symptom, the symptom was only muscular fatigability involving the limbs [14]. Oculomotor dysfunction in ALS was also previously reported [15]. However, the specific symptoms reported were pursuit movement and saccadic impairments. Myasthenic symptoms in our two patients were different from those in these previous reports. Our patients developed decreased ocular motility and showed the marked fluctuation of symptoms within a day and on a daily basis. These symptoms are uncommon in ALS and were partially improved by immunotherapies. Therefore, their myasthenic symptoms may have been partly immune-mediated.

Regarding the association between ALS and other autoimmune disorders, Turner et al. reported that the risk of ALS was increased in people with prior autoimmune diseases [16]. In addition, several autoantibodies have been detected in the serum of ALS patients and they may modify neurological symptoms of ALS [17–19]. Although our patients did not have prior symptoms suggestive of MG, it is possible that they had subclinical immunodeficiency and anti-LRP4 antibody may have caused additional symptoms after the onset of ALS in the context of the progression of axonal loss. Of further relevance, the co-occurrence of ALS and MG is rare but can develop [20, 21]. In those reports, some patients developed myasthenic symptoms after the onset of ALS symptoms, similarly to our patients, although they were anti-AChR antibody-seropositive. These findings indicate that a common immunological pathway involving the NMJ may influence the clinical course of some ALS patients.

LRP4 is one of the essential components of the NMJ, and it binds to neural agrin and MuSK protein, leading to AChR clustering and muscle excitation [22]. The pathogenesis of anti-LRP4 antibody has been studied in the presence of MG, and its detection rate in double-seronegative MG patents was 9.4% in a previous study with a large cohort [23]. On the other hand, Tzartos et al. reported that anti-LRP4 antibody was detected in the serum and CSF of sporadic ALS patients at 23.4% of the detection rate in the serum of ALS patients [10]. Consequently, the pathological significance and mechanism of production of anti-LRP4 antibody in neuromuscular disorders remain unclear. However, based on the previous study by Shen et al. [9], anti-LRP4 antibody may cause immunotherapy-responsive myasthenic symptoms in ALS patients.

In ALS, some experimental and pathological studies reported that NMJ degeneration was observed in the earliest stage of the disease, and this change may be the origin of the disease pathogenesis [3, 24]. As such, the clinical courses of our two patients indicate that an anti-LRP4 antibody-seropositive status may influence developing ALS and occasionally cause additional symptoms in the form of myasthenic symptoms.

## Abbreviations

A.I.: Antibody index
AChR: Acetylcholine receptor
AH: Abductor halluces
ALS: Amyotrophic lateral sclerosis
APB: Abductor pollicis brevis
EDC: Extensor digitorum communis
EMG: Electromyography
FDI: First dorsal interossei
FPs: Fasciculation potentials
LRP4: Low-density lipoprotein receptor-related protein 4
MEP: Motor-evoked potential
MG: Myasthenia gravis
MRC: Medical Research Council
MRI: Magnetic resonance imaging
MuSK: Muscle-specific kinase
NCS: Nerve conduction study
NMJ: Neuromuscular junction
QMG: Quantitative myasthenia gravis
RNST: Repetitive nerve stimulation testing
s-SFEMG: Stimulated-single fiber electromyography
TA: Tibialis anterior
